# Improvement of Aroma in Transgenic Potato As a Consequence of Impairing Tuber Browning

**DOI:** 10.1371/journal.pone.0014030

**Published:** 2010-11-17

**Authors:** Briardo Llorente, Vanina Rodríguez, Guillermo D. Alonso, Héctor N. Torres, Mirtha M. Flawiá, Fernando F. Bravo-Almonacid

**Affiliations:** 1 Instituto de Investigaciones en Ingeniería Genética y Biología Molecular (INGEBI), Consejo Nacional de Investigaciones Científicas y Técnicas (CONICET), Buenos Aires, Argentina; 2 Departamento de Fisiología, Biología Molecular y Celular, Facultad de Ciencias Exactas y Naturales, Universidad de Buenos Aires, Buenos Aires, Argentina; 3 Departamento de Ciencia y Tecnología, Universidad Nacional de Quilmes, Bernal, Buenos Aires, Argentina; Claremont Colleges, United States of America

## Abstract

Sensory analysis studies are critical in the development of quality enhanced crops, and may be an important component in the public acceptance of genetically modified foods. It has recently been established that odor preferences are shared between humans and mice, suggesting that odor exploration behavior in mice may be used to predict the effect of odors in humans. We have previously found that mice fed diets supplemented with engineered nonbrowning potatoes (-PPO) consumed more potato than mice fed diets supplemented with wild-type potatoes (WT). This prompted us to explore a possible role of potato odor in mice preference for nonbrowning potatoes. Taking advantage of two well established neuroscience paradigms, the “open field test” and the “nose-poking preference test”, we performed experiments where mice exploration behavior was monitored in preference assays on the basis of olfaction alone. No obvious preference was observed towards -PPO or WT lines when fresh potato samples were tested. However, when oxidized samples were tested, mice consistently investigated -PPO potatoes more times and for longer periods than WT potatoes. Congruently, humans discriminated WT from -PPO samples with a considerably better performance when oxidized samples were tested than when fresh samples were tested in blind olfactory experiments. Notably, even though participants ranked all samples with an intermediate level of pleasantness, there was a general consensus that the -PPO samples had a more intense odor and also evoked the sense-impression of a familiar vegetable more often than the WT samples. Taken together, these findings suggest that our previous observations might be influenced, at least in part, by differential odors that are accentuated among the lines once oxidative deterioration takes place. Additionally, our results suggest that nonbrowning potatoes, in addition to their extended shelf life, maintain their odor quality for longer periods of time than WT potatoes. To our knowledge this is the first report on the use of an animal model applied to the sensory analysis of a transgenic crop.

## Introduction

Olfactory perception is an advantageous evolutionary mechanism strongly shaped by experience and learning [1] that exerts a great influence in animals lives [2], including food intake [3]. However, recent findings indicate that olfactory preferences are also partially innate [1] and conserved across mammalian species [2]. Considering that prediction of the perceptual properties of novel odorants by its chemical structure is extremely difficult [1], [4], analysis of mice investigation time (when smelling an attractive odor, mice spend more time investigating the odorant source than when encountering a less attractive odor [2]) has been proposed to be a useful mean to predict human olfactory preferences [2].

Sensory analysis studies are mandatory in the development of any new food and are critical in the development of quality enhanced transgenic crops [5]. Additionally, they may be an important component in the public perception and acceptance of genetically modified foods [5], especially considering the consumers growing demand for enhanced quality characteristics in food [6]. Potato polyphenol oxidases [PPO; EC (Enzyme Commission) 1.14.18.1 or EC 1.10.3.1] are the enzymes responsible for the enzymatic browning reaction observed in impacted, damaged or sliced tubers [7]. This phenomenon is caused by the encounter of PPO and vacuolar phenolic compounds after tissue damage takes place and the subsequent PPO-catalyzed oxidation of these compounds that cross-react and precipitate as dark-colored melanin-like polymers [7]. These oxidative deterioration reactions alter organoleptic properties of food and greatly affect potato tuber quality [8], [9]. We recently reported the generation of genetically engineered potato plants with silenced polyphenol oxidase transcripts and reduced PPO enzymatic activity, resulting in the obtainment of nonbrowning potatoes [10]. These modified plants (-PPO) presented yield-associated traits and photosynthesis parameters equivalent to those of wild-type (WT) control plants, without perturbed growth or development, under our controlled conditions [10]. However, we found several alterations in the primary metabolism of -PPO tubers that may affect their organoleptic properties. In accordance with this speculation, we found that mice consumed significantly (*P≤0.05*) more -PPO potato (∼11%) than WT potato when feeding trials were performed [10]. Considering the metabolic alterations found in the -PPO potato tubers, their resistance to oxidative deterioration, and that perception of the smell of a food precedes its ingestion and also the perception of its flavor [3], we decided to explore a possible role of potato odor in mice preference for nonbrowning potatoes.

Recently, the dilemma of determining animal odor preferences was approached by the analysis of nose-poking in mice [2], [11]. However, this type of approach does not measure preference between two samples, since each type of food/odor is tested separately and the animal is not offered any food choice [12]. Therefore, we used two neuroscience paradigms, the “open field test” [13], [14] and the “nose-poking preference test” [2], [11], and performed experiments where mice exploration behavior was monitored in preference assays on the basis of olfaction alone. Additionally, we performed sensory analyses in humans [15], [16] with WT and -PPO potato samples in two blind olfactory experiments. To our knowledge, this is the first report applying the fundamental principles announced by Mandairon *et al*. [2] and is also the first time that an animal model is applied to the sensory analysis of a transgenic crop.

## Results

### Validation of the open field olfactory preference assay

**Figure 1:**
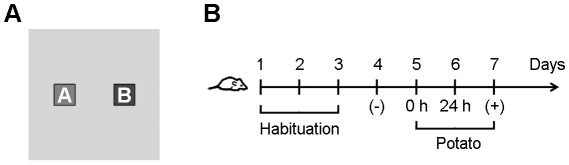
Mouse open field experimental design. (A) Schematic upper view of the open field activity boxes with zones A and B comprising food containers. (B) Diagram showing the open field experimental procedure. From day 1 to day 3 mice were habituated to the activity boxes with empty food containers. On day 4, mice exploration behavior was monitored with empty food containers and the data obtained was considered as a negative experimental control (−). On day 5, freshly cut potato samples (0 h) were randomly placed in containers A or B. On day 6, oxidized potato samples (24 h) were placed in the opposite positions with respect to day 5. On day 7, freshly cut WT potato samples were placed in one container while the other container remained empty and the data obtained was considered as a positive experimental control (+).

**Figure 2:**
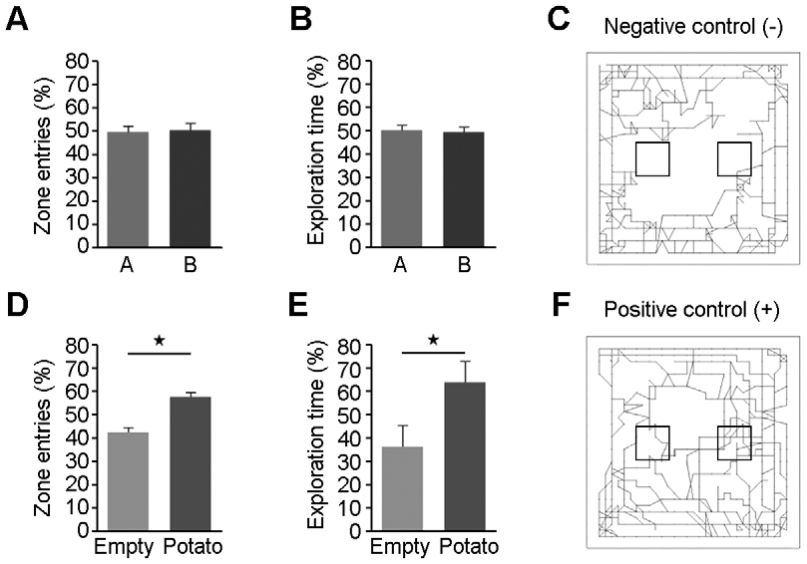
Mouse open field test experimental validation. Percentage of entries (A) and exploration time spent (B) by mice in zones A and B of the open field with both food containers empty. (C) Representative 5 min mouse trajectory of a negative experimental control where both food containers were empty. Percentage of entries (D) and exploration time spent (E) by mice in zones A and B of the open field with one container filled with freshly cut WT potato and the other one left empty. (F) Representative 5 min mouse trajectory of a positive experimental control where food container A was empty and food container B was filled with freshly cut WT potato samples. Central darker squares in Figures C and F represent zones A (on the left) and B (on the right). Stars represent statistically significant differences (*P≤0.01*) according to the one-sample *t*-test for difference from 50%. Error bars represent the ±95% confidence interval of eight independent experiments.

One of the most common models to study exploratory behavior in laboratory animals is the open field test [13]. The activity box is an open field that allows the quantification of the animals mobility and exploratory behavior without the interference from an experimental observer [13]. Recently, it has been established that this technique can be used to determine odor preferences in rodents [14]. Therefore, we designed a modified two-choice preference assay similar to that described by Smith *et al*. [14] that includes two identical food containers equidistantly positioned in each activity box (Fig. 1A). These food containers were specifically designed to avoid physical contact and the experiments were conducted in complete darkness to assure that the mouse's interest for the experimental samples was motivated on the basis of olfaction alone. A scheme detailing the experimental procedure is depicted in Figure 1B. Analysis of mice exploration behavior demonstrated that mice had no preference for either food container locations (A or B) when both food containers were empty (Fig. 2A and 2B). Additionally, as exemplified in Figure 2C, mice avoided crossing the central area and instead preferred the periphery and, particularly, the corners. On the other hand, when one container was filled with fresh WT potato samples and the other one was empty, mice always showed an increased interest for the potato-filled container (Fig. 2D–F). Considering these results, we concluded that the experimental design was useful for the identification of odors evoking attraction responses in mice, thereby allowing the discrimination of mice odor preferences.

### Mice present odor preferences for nonbrowning potatoes when oxidative deterioration occurs

**Figure 3:**
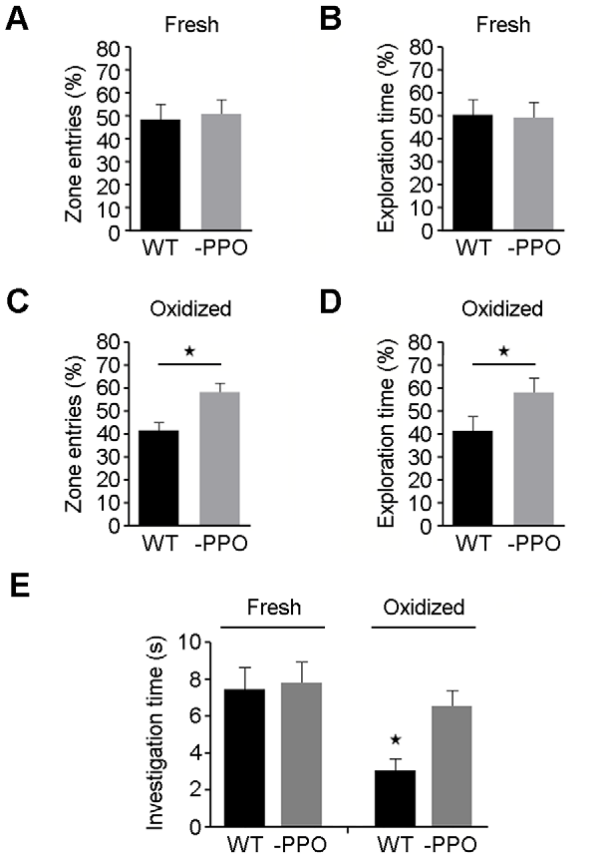
Odor exploration behavior in mice. Percentage of entries (A) and exploration time spent (B) by mice in zones A and B of the open field with food containers filled with freshly cut (0 h) WT or -PPO potato samples. Percentage of entries (C) and exploration time spent (D) by mice in zones A and B of the open field with food containers filled with oxidized (24 h) WT or -PPO potato samples. Stars represent statistically significant differences (*P≤0.03*) according to the one-sample *t*-test for difference from 50%. Error bars represent the ±95% confidence interval of eight independent experiments. (E) Hole-board experiment. Mean investigation times (s) ± SEM of six independent measurements are shown for each type of sample. The star represents statistically significant differences (*P≤0.05*) in investigation time according to the ANOVA test followed by the Newman-Keuls multiple comparison post-hoc test.

Having established that the experimental design was suitable to discriminate mice odor preferences, we next analyzed the data obtained for WT and -PPO tuber lines with our paradigm. Behavioral analyses demonstrated that mice had no exploratory preference for either WT or -PPO freshly cut (0 h) potato sample locations (Fig. 3A and 3B). However, when these experiments were repeated with partially oxidized (24 h) WT and -PPO samples, mice consistently presented a greater interest for the -PPO potatoes over the WT potatoes. These results were statistically significant (*P≤0.03*) according to analysis by the one-sample *t*-test for difference from 50% (random exploration) (Fig. 3C and 3D). While there was no significant difference from the null expectation for the freshly cut potato samples, there was a significant difference from the null expectation for the oxidized potato samples, suggesting that dissimilar odors are accentuated between potato lines once oxidative deterioration takes place. Additionally, to confirm our open field results, we performed a nose-poking preference experiment using a hole-board apparatus as previously described [2], [11]. Mice demonstrated significantly (*P≤0.05*) reduced exploration times according to the ANOVA and subsequent *ad hoc* Newman-Keuls tests only with WT oxidized (24 h) samples, as shown in Figure 3E. These results suggest that nonbrowning potatoes maintain their odor quality for longer periods of time than WT potatoes.

### Humans are able to discriminate transgenic from wild-type potato samples on the basis of their smell

**Figure 4:**
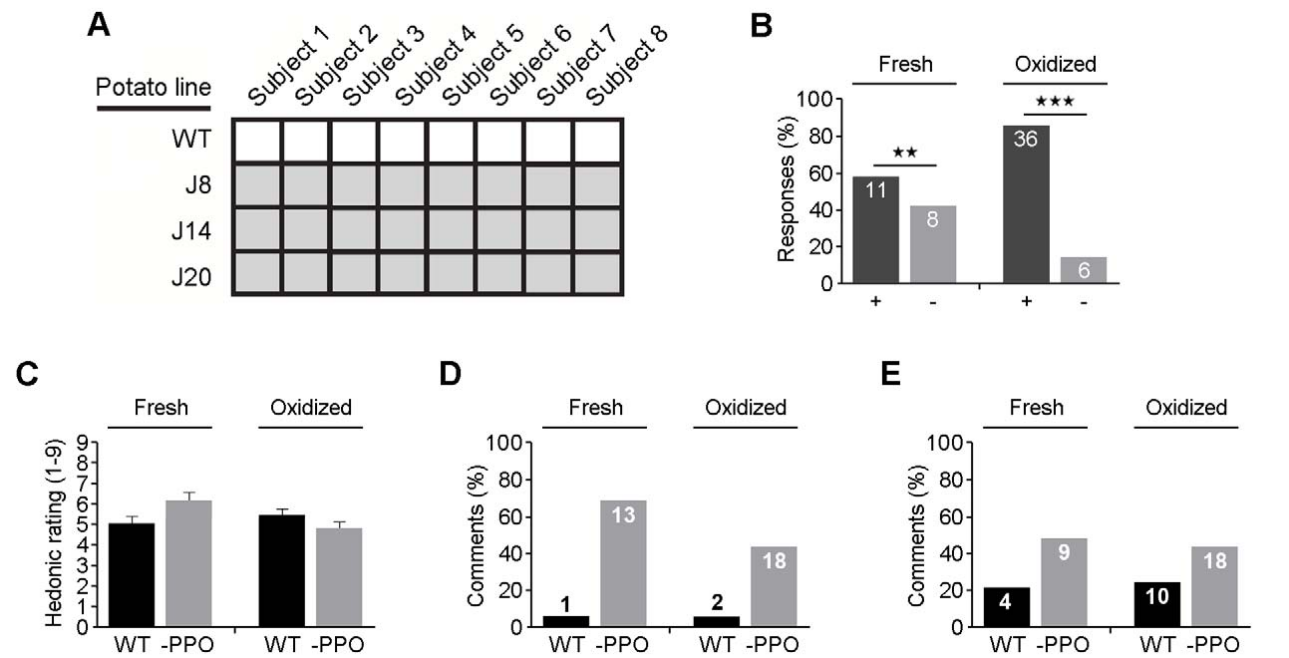
Humans sensory analyses. (A–B) Smell discrimination by humans. (A) Smell discrimination with all transgenic lines. A filled box indicates that the odor of the sample was described as more intense by the subject. (B) Triangle test with fresh and oxidized samples. The number of correct answers to be significant is 11 (*P≤0.03*) correct responses for the group tested with fresh samples (n = 19) and 25 (*P≤0.001*) correct responses for the group tested with oxidized samples (n = 42). The number of correct responses (+) was determined by counting the number of participants that chose the unique sample of the three. The number of incorrect responses (−) equals the number of participants not choosing the distinct sample of the three. The percentage of correct and incorrect responses is depicted and the number of the corresponding responses is shown inside each bar. Two stars represents statistically significant differences at *P≤0.03* and three stars represents statistically significant differences at *P≤0.001*. (C–E) Organoleptic evaluations. (C) Hedonic rating. Being 1: “not at all pleasant” and being 9: “very pleasant”. No statistical differences were found among samples according to the ANOVA test. (D) Comments describing that the odor of the sample was more intense. The percentage of comments is depicted and the number of the corresponding comments is shown inside or above each bar. (E) Comments describing that the odor of the sample evoked the sense-impression of a familiar vegetable. The percentage of comments is depicted and the number of the corresponding comments is shown inside or above each bar.

To further investigate if the odor differences between oxidized transgenic and wild-type potatoes were also discriminated by humans an additional set of experiments with untrained panelists were performed [15], [16]. We first tested if humans were able to discriminate oxidized samples from WT and three -PPO lines (J8, J14 and J20). In this preliminary experiment, 100% of the evaluators unequivocally rated -PPO samples as more odoriferous than WT samples (Fig. 4A) and overall, the participants commented positively on the odor of transgenic tubers. Subsequently, a second experiment with 61 participants was performed, testing one group of participants (n = 19) with fresh samples and a second group of participants (n = 42) with oxidized samples. In this larger experiment, we confirmed that humans could perceive differences between the odors from the WT and -PPO samples and that these differences were discriminated with a considerably better performance by the group tested with oxidized samples. The group tested with fresh samples presented a 57.89% of correct responses, which was significant at a *P*-value of *P≤0.03* and the group tested with oxidized samples presented an 85.71% of correct responses, which was significant at a *P*-value of *P≤0.001* (Fig. 4B). Notably, even though participants ranked all samples with an intermediate level of pleasantness (4.81–6.15) and the differences were not significant (Fig. 4C), there was a general consensus that the -PPO samples had a more intense odor (Fig. 4D) and also evoked the sense-impression of a familiar vegetable more often than the WT samples (Fig. 4E).

## Discussion

A new wave of genetically engineered crops displaying enhanced quality traits is being developed [5], [15], [17]–[22]. It is expected that these new crops will provide perceivable benefits to both farmers and consumers, thus improving public perception of genetic engineering technology [23]. Additionally, enhanced quality characteristics in food may encourage consumers' adoption and thus have a beneficial effect in both human nutrition and agricultural business [5]–[23]. The application of sensory evaluation studies in the development of genetically modified foods could help in determining whether these novel products are commercially viable [5].

With the aim of improving potato quality, we engineered plants to produce potatoes with reduced tuber browning [10]. While performing feeding experiments, we found that mice fed diets supplemented with these nonbrowning potatoes consumed more potato than mice fed diets supplemented with wild-type potatoes [10]. Our initial interpretation of these results was that this could originate from the differential organoleptic properties between -PPO and WT tubers arising from metabolic dissimilarities and/or due to the reduced browning in -PPO tubers, allowing a slower deterioration and making them palatable for longer periods of time than WT tubers [10]. However, since perception of the smell of a food precedes and highly influences its ingestion [3], in the present study, we explored a possible role of potato odor in mice preference for nonbrowning potatoes.

Although it is well established that polyphenol oxidases catalyze oxidative reactions that alter the organoleptic properties of food [8],[9], the significance of impairing the enzymatic browning on potato tuber aroma has not been previously studied. By using a combination of mouse behavioral paradigms and human sensory evaluations, we show that impairing the oxidative reactions catalyzed by PPO alters potato tuber smell. The evident preference of mice for -PPO potatoes when oxidized samples were tested seems to indicate that, regardless of any possible initial odor difference between the lines, transgenic tubers conserve an increased amount of key volatiles that contribute to potato smell for extended periods of time. This is also indicated by the results of the human sensory analyses, where subjects were able to discriminate WT from -PPO samples with a better performance when the samples were oxidized, supporting our results obtained in mice. The lack of significant differences in the hedonic rating test may be explained by the fact that human evaluation of smells is particularly subjective because of interspecific variations in the ability to detect and describe smells [24] and could also be a consequence of the use of untrained panelists. Additionally, the odor of raw potatoes may not represent a significantly pleasant stimulus to humans, as the intermediate scores given to all samples seem to indicate. However, it is worth mentioning that participants evoked the sense-impression of a familiar vegetable more often when smelling the transgenic samples than when smelling the WT samples.

Although the molecular basis ruling the differential odor between WT and -PPO lines was not studied as part of this work, several metabolites that contribute to smell are altered in the transgenic lines. For example, myristic acid, which is used in the manufacture of perfumes and flavorings and has been shown to be an odor attractant for *Anopheles gambiae* [25] is over twofold increased in the transgenic lines [10]. Furthermore, the amino acid pools of phenylalanine, methionine, valine, leucine and isoleucine, which are known precursors of volatile compounds in plants [26], [27] are also altered in the transgenic lines [10]. These alterations may be indicative of a metabolic shift towards the production of volatile compounds. Additionally, it can be speculated that the inhibition of the enzymatic browning reaction may prevent the polymerization and subsequent precipitation of volatile phenols, or other volatile compounds [27], therefore remaining free and contributing to tuber smell. In line with this speculation, previous studies have shown that not only the odor quality, but also the odor intensity (odorant concentrations) can influence olfactory preferences [11], [28]–[31]. Further profiling studies of the volatile molecules in the -PPO potatoes will provide an additional level of knowledge to better interpret our results and will help in the quest for the development of new improved cultivars.

Taken together, our findings suggest that our previous observations - that mice consume more -PPO potato than WT potato [10] - might be influenced, at least in part, by differential odors that are accentuated among the lines once oxidative deterioration takes place. Additionally, our results suggest that nonbrowning potatoes, in addition to their extended shelf life, maintain their odor quality for longer periods of time than WT potatoes.

## Materials and Methods

### Ethics statement

All animal and human experimental procedures were approved by the Bioethics Committee of the Instituto de Biología y Medicina Experimental (IBYME-CONICET; Bioethics Committee approval resolution 11-01-2010) in accordance to the declaration of Helsinki and the Argentinean National Research Council Directive RD_N° 1047/05 for ethical research. All participants gave written consent after receiving a full explanation of the study.

#### Ethics Committee statement

In reference to the project entitled “Olfactory preference study in nonbrowning potatoes”, presented for evaluation to this Committee. The Ethics Committee found no objections to the experimental procedures described in this project. International normative should be followed and written consent should be obtained.

### Potato samples

WT and -PPO [10] potato plants (*Solanum tuberosum* var. Spunta) were grown in 4-L pots under greenhouse conditions (25±3°C and 16 h light/8 h dark photoperiod). Tubers were harvested when the plants senesced after growth for approximately four months. Three -PPO lines, J8, J14 and J20, with reduced levels of polyphenol oxidases were used in the human sensory analysis. Line J14, showing the highest inhibition of PPO enzymatic activity among the transgenic lines [10] was selected for the mice odor preference assays. Experimental samples consisted of 2 g (for the open field experiments), 3 g (for the hole-board experiments), 80 g (for the first human experiments) or 20 g (for the second human experiments) of freshly cut (0 h) or oxidized potato tuber disks (8-mm-diameter×1-mm-thick). Oxidized samples consisted of samples placed in hermetic plastic bags and maintained for approximately 24 h (for mouse experiments) or 4–6 h (for human experiments) at room temperature in the dark.

### Animals

Studies were carried out using a total of 32 8-week-old female BALB/c mice (Animal Facility of the School of Veterinary Sciences Faculty, National University of La Plata, La Plata, Argentina). Eight mice were used in the open field experiments and 24 mice in the hole-board experiments. Animals were housed and kept under standard conditions (12 h light-dark cycle, air-conditioned room, 21±1°C, relative humidity 60±10%) in accordance with the guide for the Care and Use of Laboratory Animals of the Public Health Service (USA). Mice were housed in groups of four per cage (31×22×16 cm) to improve animals' welfare. Rodent pellet food (GEPSA Feeds, Argentina) and water were available *ad libitum* except during behavioral testing. After acclimatization for 7 days, mice were subjected to the behavioral studies.

### Mice behavioral studies

All experiments were performed during the dark period, between 19:00 h and 22:00 h under complete darkness, in a separated behavioral room where mice were habituated for 3 days in advance. Four acrylic open field activity boxes (Med Associates Inc., St Albans, VT, USA) coupled to a computer interface were used to assess horizontal, vertical and stereotyped activity. Two identical plastic food containers (35-mm-diameter×12-mm-thick) specifically designed to avoid physical contact with the food (food was separated by a metallic net) were equidistantly positioned inside each open field activity box. Food containers were secured to the open-field floor with tape. Animals were placed in one of the four activity boxes (40×40×40 cm) and horizontal and vertical activities were measured by disruption of infrared beams separated by 2.5 cm that cross the x–y plane at two z-levels. Stereotyped behavior was measured by repetitive disruptions of single infrared beams. Open field activity was performed using a protocol consisting of 5-min adaptation and a trial duration of 15 min [32]. Two analysis zones (6×6 cm), designated A and B (see Figure 1A), covering the positions of each container were generated with the computer and the number of entries and exploration time spent by mice in zones A and B were considered. The number of entries and the duration of exploration time in the zones were used as a measure of odor preference in a similar fashion to previously described methods [14], [29]. From day 1 to day 3 mice were habituated to the activity boxes with empty food containers for 60 min. On day 4, mice exploration behavior was monitored with empty food containers and the data obtained was considered as a negative experimental control. On day 5, fresh potato samples (0 h) were randomly placed in container A or B and food position was inverted on day 6 (24 h =  oxidized samples), such that if WT and -PPO samples were placed in containers A and B respectively at 0 h, they were then placed in containers B and A respectively at 24 h. On day 7, fresh WT potato samples were placed in the opposite container occupied by -PPO samples on day 6 while the other container remained empty. Data obtained on day 7 was considered as a positive experimental control. The positive experimental control was deliberately performed on day 7, after testing the 0 h and 24 h WT and -PPO samples to avoid mice preference habituation to either of the two food containers locations. Additional controls with both containers filled with freshly cut WT potato samples and one container filled with pellet food and the other one left empty were performed on day 8 and day 9 respectively (data not shown) and results were comparable to those obtained in day 4 (negative experimental control) and in day 7 (positive experimental control) respectively. An additional set of experiments were performed using a one-hole-board apparatus (40×40×40 cm) constructed as previously described [11], with the addition of opaque walls to improve animals' performance during the task. The sequence and duration of mice nose poking into the hole was monitored under infrared light with a digital infrared video camera for the analysis [29]. Mice were habituated for three days to the hole-board apparatus for 30 min and the following day, six mice for each type of sample were used in the test [29]. To avoid data owing to learning, mice were used only once [29]. Each trial lasted 2 min [2], [11] and duration (seconds) of nose poking into the hole was used as a measure of odor preference [2], [11], [33]. Boxes, food containers and the hole-board apparatus were carefully cleaned between tests to minimize odor cues in the arenas.

### Sensory evaluation in humans

A total of 69 healthy volunteers (20 to 50 years old) recruited from the Instituto de Investigaciones en Ingeniería Genética y Biología Molecular participated in two blind olfactory experiments [15], [16]. Participants were asked to close their eyes while performing the experiment. Additionally, subjects' eyes were covered during the experiment to avoid visual perception. In the first experiment, eight subjects were asked to smell a set of unidentified samples and to estimate their odor intensities. Samples were presented on petri dishes containing 80 g of WT or -PPO (J8, J14 or J20) potato tuber disks (8-mm-diameter×1-mm-thick). Three independent samples of every potato line were presented to each subject. Samples were randomly presented every 45 seconds. In the second experiment, a triangle test was performed [34] with 61 subjects divided into two groups. The first group (19 subjects) was tested with fresh potato samples while the second group (42 subjects) was tested with oxidized potato samples. Each subject was randomly given a set of 3 unidentified samples that consisted of two samples of the same genotype and one of the other genotype. The test was performed both with two controls (WT) and one transgenic (only line J14 was used in this experiment) or two transgenics and one control. Samples were presented on identical opaque vials containing 20 g of WT or -PPO potato tuber disks (8-mm-diameter×1-mm-thick). Subjects were asked to judge which sample had a different odor [34], and to rate, using an arbitrary scale of 1 to 9 (being 1: “not at all pleasant” and being 9: “very pleasant”), the pleasantness of the samples [2], [15]. Additional descriptive comments were also solicited from the participants [15], [34] in both human experiments.

### Data analysis

Mouse behavioral data from the open field experiments were analyzed using the Activity Monitor Software Version 5 (Med Associates Inc., St Albans, VT, USA). The percentage of entries and exploration time spent by mice in zones A and B of the open field were calculated by considering the total number of entries or time spent in zones A plus B as 100%. Statistical significance was determined by the one-sample *t*-test for difference from 50% analysis. The null hypothesis was that 50% of the times mice should visit and stay in either of the two experimental zones and a significant deviation from 50% indicated a significant preference [35]. The ANOVA and the Newman-Keuls multiple comparison post-hoc tests were used to determined statistical significance in the hole-board [11] and the human hedonic rating experiments. The statistical significance of the correct number of judgments in the triangle test was determined using the tables described in [36]. A response was considered correct when a subject assigned a different odor to the genotype that was represented by a single sample. Statistical analysis was performed using GraphPad Prism 5 (GraphPad Software, USA).
